
JOURNAL INFORMATION
==============================
NLM Title Abbreviation: Wirtschaftsdienst
Journal ID: Wirtschaftsdienst
NLM Journal ID: 101086612

Wirtschaftsdienst (Hamburg, Germany : 1949)

ISSN: 0043-6275

ARTICLE INFORMATION
==============================
PMCID: PMC7237611
PMID: 32454548
DOI: 10.1007/s10273-020-2661-z
Article ID: 2661
Article version: 1
Subjects: Ökonomische Trends

Konjunkturschlaglicht: Weltweiter Corona-Schock am Arbeitsmarkt

Business cycle highlight: Global corona shock on the labour market

Gern Klaus-Jürgen klaus-juergen.gern@ifw-kiel.de
1
Hauber Philipp philipp.hauber@ifw-kiel.de
2
Stolzenburg Ulrich ulrich.stolzenburg@ifw-kiel.de
3
1 grid.462465.7 0000 0004 0493 2817 Forschungsgruppe Intern. Konjunktur, Institut für Weltwirtschaft, Kiellinie 66, 24105 Kiel, Deutschland
2 grid.462465.7 0000 0004 0493 2817 Prognosezentrum, Institut für Weltwirtschaft, Kiellinie 66, 24105 Kiel, Deutschland
3 grid.462465.7 0000 0004 0493 2817 Institut für Weltwirtschaft, Kiellinie 66, 24105 Kiel, Deutschland
Electronic publication date: 2020 May 20
Print publication date: 2020
Volume: 100
Issue: 5
First page: 387
Last page: 388
Copyright: © Der/die Autor(en) 2020
License: Open Access Dieser Artikel wird unter der Creative Commons Namensnennung 4.0 International Lizenz veröffentlicht, welche die Nutzung, Vervielfältigung, Bearbeitung, Verbreitung und Wiedergabe in jeglichem Medium und Format erlaubt, sofern Sie den/die ursprünglichen Autor(en) und die Quelle ordnungsgemäß nennen, einen Link zur Creative Commons Lizenz beifügen und angeben, ob Änderungen vorgenommen wurden.
Die in diesem Artikel enthaltenen Bilder und sonstiges Drittmaterial unterliegen ebenfalls der genannten Creative Commons Lizenz, sofern sich aus der Abbildungslegende nichts anderes ergibt. Sofern das betreffende Material nicht unter der genannten Creative Commons Lizenz steht und die betreffende Handlung nicht nach gesetzlichen Vorschriften erlaubt ist, ist für die oben aufgeführten Weiterverwendungen des Materials die Einwilligung des jeweiligen Rechteinhabers einzuholen.
Weitere Details zur Lizenz entnehmen Sie bitte der Lizenzinformation auf http://creativecommons.org/licenses/by/4.0/deed.de.
License URL: https://creativecommons.org/licenses/by/4.0/

issue-copyright-statement: © The Author(s) 2020

==============================

COVID-19 hat die Weltwirtschaft im Frühjahr 2020 fest im Griff. Ausgehend von China hat sich die Pandemie im Februar und März weltweit ausgebreitet, und inzwischen sind praktisch überall Maßnahmen zur Eindämmung des neuartigen Coronavirus ergriffen worden, welche die wirtschaftliche Aktivität massiv einschränken. Die konjunkturellen Auswirkungen sind dramatisch; zu erwarten ist der stärkste Einbruch der Weltproduktion seit dem Zweiten Weltkrieg. Der Internationale Währungsfonds (2020) rechnet mit einem Rückgang im laufenden Jahr 2020 um 3 %, in den meisten fortgeschrittenen Volkswirtschaften dürfte das Bruttoinlandsprodukt sogar noch erheblich stärker sinken.

Der Konjunktureinbruch wirkt sich auf dem Arbeitsmarkt in einem drastischen Rückgang der Beschäftigung aus. Nach Schätzungen der Internationalen Arbeitsorganisation (2020) wird sich die Zahl der geleisteten Arbeitsstunden im Jahr 2020 weltweit um 6,7 % verringern, was einem Vollzeitäquivalent von 230 Mio. Beschäftigten entspricht. Der Grad der Produktionseinschränkung unterscheidet sich je nach Wirtschaftsbereich; besonders stark betroffen sind personenbezogene Dienstleistungen wie das Gastgewerbe und die Unterhaltungsindustrie, Einzelhandel oder Transportdienstleistungen, aber auch das Verarbeitende Gewerbe, wo neben staatlich verordneten Produktionsstillständen auch Probleme in den Lieferketten die Produktion beeinträchtigen.

Auf Unternehmensebene ist ein drastischer Abbau des Arbeitseinsatzes erforderlich, um die Kosten an die gesunkenen Einnahmen anzupassen und so Verluste zu begrenzen. Damit ein vorübergehender Rückgang der Produktion nicht unmittelbar zu Entlassungen in entsprechendem Umfang führt, sind in vielen Ländern Regelungen vorgesehen, die es erlauben, Arbeitskräfte unter Beibehaltung des Beschäftigungsverhältnisses ganz oder teilweise freizustellen, wobei der Staat Lohnersatzleistungen zahlt. In Deutschland hat das Kurzarbeitergeld eine lange Tradition, aber auch in vielen anderen Ländern der Organisation für wirtschaftliche Zusammenarbeit und Entwicklung (OECD), vor allem in Europa, wurden entsprechende Instrumente bereits im Verlauf der Großen Rezession 2008/2009 eingesetzt. Als Reaktion auf die COVID-19-Krise wurden sie in weiteren Ländern eingeführt, etwa in Großbritannien, oder zum Teil erheblich ausgeweitet. Ein Beispiel für dessen Wirkung ist Österreich, wo die Arbeitslosigkeit (gemäß Arbeitsamtsstatistik) im März 2020 als Folge des teilweisen Shutdowns der Wirtschaft zunächst kräftig von 7,4 % auf 12,2 % stieg, sich im April aber nur noch moderat auf 12,8 % erhöhte, weil das Kurzarbeitsprogramm ausgeweitet und attraktiver ausgestaltet worden war. So waren Ende April mehr als 1 Mio. Beschäftigte zur Kurzarbeit angemeldet – dies entspricht etwa 30 % aller Beschäftigten –, von denen sonst wohl ein erheblicher Teil in die Arbeitslosigkeit entlassen worden wäre.

Die Möglichkeit der Kurzarbeit wird in Europa derzeit in enormem Umfang genutzt. In Deutschland sind Ende April 10 Mio. und damit nahezu ein Viertel der Beschäftigten in Kurzarbeit. Ähnlich groß ist der Anteil in Italien, in Frankreich liegt er sogar bei rund einem Drittel. Auch wenn diese Zahlen eine absolute Obergrenze der Inanspruchnahme darstellen – nur angemeldete Kurzarbeit kann auch tatsächlich abgerechnet werden – sind sie doch um ein Vielfaches höher als in der Großen Rezession. Trotz des massiven Einsatzes von Kurzarbeit steigt in Europa derzeit auch die Arbeitslosigkeit, und die Erwartungen der Verbraucher im Euroraum in Hinblick auf Arbeitslosigkeit sind auf ein ähnliches Niveau gestiegen wie 2009 (vgl. Abbildung 1).[1] Betroffen sind zunächst vor allem Sai-

1 In Italien, das von der Pandemie besonders stark betroffen wurde, konnten im April keine Daten erhoben werden. Der für den gesamten Euroraum ausgewiesene Anstieg dürfte daher noch unterzeichnet sein.

Abbildung 1

**Arbeitslosigkeitserwartungen der Verbraucher in Europa, Economic Sentiment Indicator (ESI)**

Monatsdaten, saisonbereinigt

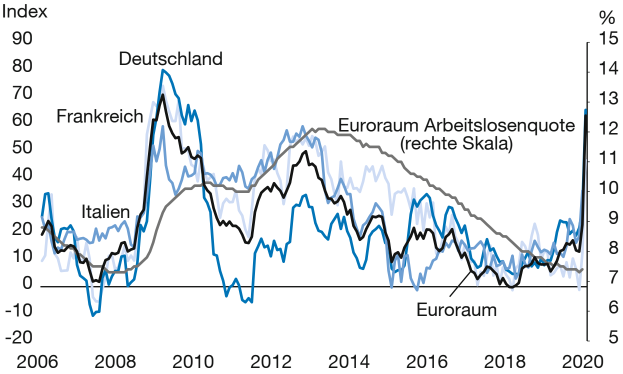

Anmerkungen: Für Italien wurde im April 2020 kein ESI-Wert ermittelt. Arbeitslosenquote für April 2020 noch nicht verfügbar.

Quelle: Europäische Kommission, Eurostat.

Abbildung 2
**Arbeitslosigkeit und Beschäftigungsquote in den USA**
Monatsdaten, saisonbereinigt

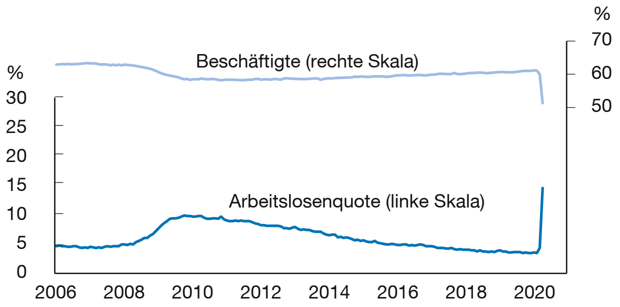

Anmerkung: Beschäftigte: im Verhaltnis zur Gesamtbevölkerung.

Quelle: Bureau of Labor Statistics, Employment Situation.

sonarbeitskräfte und Beschäftigte außerhalb der Stammbelegschaft, deren Zeitverträge nicht verlängert werden. Hinzu kommen weitere Faktoren: So war etwa der saisonbereinigte Anstieg der Arbeitslosenquote im April 2020 in Deutschland von 5,0 % auf 5,8 % in erheblichem Umfang – zu fast 40 % – darauf zurückzuführen, dass im Zuge der Seucheneindämmungspolitik arbeitsmarktpolitische Maßnahmen ausgesetzt wurden. Weitere 20 % lassen sich dadurch erklären, dass kaum noch Neueinstellungen vorgenommen wurden. Lediglich 30 % des Anstiegs geht auf Entlassungen zurück.

Ungefiltert durch das Instrument der Kurzarbeit zeigen sich die Auswirkungen der Corona-Krise am Arbeitsmarkt in den USA. Während zur Jahreswende praktisch Vollbeschäftigung herrschte, kämpft das Land mittlerweile mit Rekordarbeitslosigkeit. Bereits im März 2020 erhöhte sich die Arbeitslosenquote von 3,5 % auf 4,4 % – der kräftigste monatliche Anstieg seit Mitte der 1970er Jahre –, und dies obwohl die der Statistik zugrundeliegenden Erhebungen in der ersten Märzhälfte stattfanden, noch bevor zur Bekämpfung der Seuche in großem Umfang soziale und wirtschaftliche Restriktionen erlassen wurden. Seither haben mehr als 30 Mio. Menschen ihren Arbeitsplatz verloren und staatliche Unterstützung beantragt, und die Arbeitslosenquote sprang im April 2020 mit knapp 15 % auf das höchste Niveau seit Ende des Zweiten Weltkriegs (vgl. Abbildung 2). Dabei unterzeichnen diese Zahlen möglicherweise noch das Ausmaß der Unterbeschäftigung, denn wer seinen Arbeitsplatz verloren hat und sich angesichts der erhöhten Arbeitslosenunterstützung[2] und der andauernden Pandemie nicht sofort auf die Suche nach einer neuen Stelle macht, wird nicht als arbeitslos gezählt. Das Verhältnis der Beschäftigten zur Gesamtbevölkerung – „employment-to-population ratio" – liefert daher möglicherweise ein umfassenderes Bild der Lage am Arbeitsmarkt. Während der Wert zu Jahresbeginn noch bei 61 % stand, fiel er bis April auf 51 % und damit etwa doppelt so stark wie während der Großen Rezession, als dieser Indikator in der Spitze um rund 5 % zurückging. Zwar dürfte sich mit der für die kommenden Monate erwarteten Normalisierung des Wirtschaftslebens auch die Arbeitslosigkeit rasch wieder zurückbilden; das sehr niedrige Vorkrisenniveau wird sie aber wohl noch lange deutlich überschreiten.

Besonders dramatisch dürfte sich der Corona-bedingte Einbruch der Beschäftigung in den Entwicklungs- und Schwellenländern auswirken. Vielerorts sind dort – wohl wegen der geringeren Leistungsfähigkeit der Gesundheitssysteme – besonders strikte Ausgangssperren in Kraft gesetzt worden, die auch streng kontrolliert werden. Während in den fortgeschrittenen Volkswirtschaften die Einkommensausfälle in erheblichem Umfang durch staatliche Unterstützungszahlungen ausgeglichen werden, existieren solche Programme in den meisten Entwicklungs- und Schwellenländern außerdem nur rudimentär. Hinzu kommt, dass in diesen Ländern ein großer Teil der Arbeitskräfte im informellen Sektor beschäftigt ist, wo ohnehin keine Ansprüche auf staatliche Unterstützungsleistungen entstehen. Dies gilt auch für China, wo sich die vom nationalen statistischen Amt ausgewiesene Arbeitslosenquote zwar ungewöhnlich stark erhöhte, der Anstieg von 5 % auf knapp 6 % das tatsächliche Ausmaß der Unterbeschäftigung aber wohl stark unterzeichnen dürfte.[3]

Entscheidend dafür, wie tief und wie nachhaltig die Krise am Arbeitsmarkt ausfällt, wird letztlich sein, wie rasch die Corona-Pandemie unter Kontrolle gebracht und die wirtschaftlichen und sozialen Einschränkungen gelockert werden können. „Bleibende Schäden" am Arbeitsmarkt werden umso geringer sein, je besser es gelingt, eine große Pleitewelle abzuwenden. Auch der massive Einsatz von Kurzarbeit wird nicht verhindern, dass zunehmend vormals gesunde Unternehmen – womöglich dauerhaft – vom Markt verschwinden, je länger die Krise andauert.

Klaus-Jürgen Gern, Philipp Hauber, Ulrich Stolzenburg
Klaus-Juergen.Gern@ifw-kiel.de

2 Im Rahmen des im März 2020 verabschiedeten CARES-Act wurden neben pauschalen Zahlungen an (fast alle) Haushalte die Arbeitslosenunterstützung auf Selbstständige ausgedehnt, die Anspruchsdauer ausgeweitet und die wöchentlichen Zahlungen für einen Zeitraum von vier Monaten um 600 US-$ aufgestockt.

3 Der Anteil informeller Beschäftigung an der Gesamtbeschäftigung liegt in China und Brasilien bei rund 50 %, in Ländern wie Indien, Indonesien oder Nigeria ist er noch bedeutend höher, vgl. Internationale Arbeitsorganisation (2020, 7).


Literatur

Internationale Arbeitsorganisation (2020), ILO Monitor: Covid-19 and the world of work, Second edition, 7. April, https://www.ilo.org/wcm-sp5/groups/public/@dgreports/@dcomm/documents/briefingnote/wcms_740877.pdf (8. Mai 2020).
Internationaler Währungsfonds (2020), World Economic Outlook April 2020, The Great Lockdown, https://www.imf.org/en/Publications/WEO/Issues/2020/04/14/weo-april-2020 (6. Mai 2020).
